# Ten years of Women's Wellness research: Key lessons from conducting randomised controlled trials of a whole-of-lifestyle behavioural intervention

**DOI:** 10.1016/j.conctc.2025.101441

**Published:** 2025-02-03

**Authors:** Sarah M. Balaam, Alexandra L. McCarthy, Natalie K. Vear, Mackenzie J. Petie, Debra J. Anderson, Janine P. Porter-Steele

**Affiliations:** a Griffith Health Group Executive, Room 8.56, Ian O'Connor building (G40), Health Group Executive, Gold Coast campus, Griffith University, QLD, 4222, Australia; b School of Nursing, Midwifery and Social Work, Level 3, Chamberlain Building (35), The University of Queensland, St Lucia, QLD, 4072, Australia; c Wesley Research Institute, Level 8, East Wing, The Wesley Hospital, 451 Coronation Dr, Auchenflower, QLD, 4066, Australia; d Wesley Choices Cancer Support Centre, 451 Coronation Dr, Auchenflower, QLD, 4066, Australia; e Faculty of Health, University of Technology Sydney, 235 Jones St, Ultimo, NSW, 2007, Australia

**Keywords:** Nursing, Oncology, Breast cancer, Gynaecological cancer, Lifestyle intervention, Randomised controlled trial, Behavioural intervention, Study design

## Abstract

Many women are diagnosed with breast cancer and while the survival of this cohort has improved, their likelihood of developing treatment-related chronic conditions is considerable. Over the last 10 years, our research group has developed and refined a whole-of-lifestyle intervention, the Women's Wellness after Cancer Program (WWACP), for women who have finished treatment for primarily breast and gynaecological cancers. Culturally-specific iterations of this program were recently completed with younger breast cancer survivors (aged <50 years) living in Australia, New Zealand/Aotearoa and Hong Kong.

Over the last decade, various approaches have been used to trial the WWACP, mostly randomised controlled trials. While this methodology is considered the gold standard to determine efficacy in health and medical research, its limitations in our interventional research are apparent. In this opinion article, we discuss these limitations as well as alternative options for the appropriate testing of behavioural studies in women treated for cancer. We also discuss how the contribution of informed consumer advocates and participant consumers has influenced changes to our study designs.

## Highlights

• Lessons learned from a decade of the Women's Wellness after Cancer Program trials.
• Study design and outcomes interpreted by consumers (advocates and participants).
• Quantitative RCT methodology not ideal for assessing health behaviour interventions.
• A mixed method, non-RCT approach to evaluate intervention efficacy is suggested.
• Otherwise, non-randomised pre-post, A/B or waitlist control test designs acceptable.

Randomised controlled trials (RCTs) are the 'gold standard' to determine efficacy in health and medical research [[1], [2], [3]] and often inform clinical practice guidelines. However, our research group has identified a significant disconnect between the quantitative outcomes generated by RCTs and participants' reported qualitative experiences within our studies of health behaviour interventions. Over the last decade our research group, which created the Women's Wellness after Cancer Program (WWACP), has been dedicated to improving quality of life (QoL) outcomes for women after their cancer treatment [[4], [5], [6], [7], [8], [9], [10], [11], [12], [13], [14], [15], [16], [17]]. Our multidisciplinary e-health lifestyle interventions target modifiable behaviours over a 12-week period. These include physical activity, diet, alcohol reduction, stress, sleep, menopausal symptoms, and sexual wellbeing; with an overall aim to reduce chronic disease risk and cancer recurrence in this population [5]. The WWACP was originally designed for women following treatment for breast, gynaecological, and/or haematological cancers. We have also undertaken several iterations of the WWACP research program with women with different chronic conditions (e.g., diabetes, heart disease, stroke and irritable bowel syndromes) and in different countries. As a result of consistent participant feedback, we have modified our study designs over time to make it easier for women to participate in our studies. Refinements range from the adoption of consumer-driven (advocates and participants) and approved outcomes and outcome measures (e.g., body image, sexuality), adaptations of the intervention to ensure cultural and language sensitivities are accommodated, reduced survey burden, and data collection methods from paper-based to automated electronic delivery. Despite these modifications, it is consistently apparent that the ‘gold standard’ of RCT design underpinning all our studies (repeatedly reported as so according to the literature [[1], [2], [3]]) is not the best fit for our research problem nor our research cohorts. Specifically, when a quantitative RCT is the selected design for a tailored health behaviour intervention that addresses multiple lifestyle factors, dissonance in outcomes and their interpretation become apparent—which can only be attributed to the nature of the quantitative randomised controlled design. Combined formal and informal consumer and research intervention team input further brought about need for adaptation of the trial format (not just program and/or intervention modifications). In this paper, we discuss the ways that the strictures of RCT affect our study outcomes and offer potential solutions.

The primary issue we encounter is the misalignment between outcomes reported quantitatively and the experiences of women in the studies reported qualitatively. Behavioural interventions such as the WWACP provide participants with knowledge, techniques and strategies designed to promote beneficial health behaviour and thereby reduce chronic disease risk. However, as cancer treatment affects every individual in a different way in every domain of health (i.e., physiologically, psychologically, and socially) and has different ramifications in the short and long terms, our intervention is always carefully-tailored to the needs, health goals and contexts of each woman. It is therefore inherently difficult to standardised outcome measures for an intervention that is not a one-size-fits-all. This is particularly difficult when intervention fidelity (i.e. the extent to which delivery of an intervention adheres to the protocol or program model originally developed) is essential for an effective RCT [18]. For instance, one of the primary outcome measures across all WWACP projects is waist circumference, taken at 80-cm target as per World Health Organisation recommendations [19], and accepted by funding bodies as a measurable and desirable outcome with which to power a study. Waist circumference reflects abdominal fat and is a good indicator of health risk. Yet the standardised 80-cm waist measurement is not safe for some women after their cancer treatment, nor is it an outcome that every participant desires or sets to change within the intervention. In addition, some participants commence additional (and allowable) treatments during the study period, such as aromatase inhibitors, which can increase menopausal symptoms and exacerbate increases in waist circumference [20]. This factor, which would be independent of the intervention and not experienced by every participant, could have a significant effect on mean within-group waist circumference. Therefore, despite no changes or even increases in objective measures such as waist circumference, the intervention might have otherwise been deemed successful via self-reported improvements in other areas such as menopausal symptom reduction, sleep quality, or overall QoL. Similar behavioural interventions in women who are obese, have chronic pain, substance abuse, or depression report parallel issues in their quantitative results, due to the individualised approach necessary to treat the specific needs and evolving status of each person [21], which are also difficult (if not impossible) to standardise via RCT principles.

Planner and colleagues [22] highlighted that RCTs often do not consider the participant's individual imperatives, which should be a key factor when trialling a behavioural lifestyle intervention that addresses multiple, usually intertwined, lifestyle factors. In the WWACP, formalised participant focus group discussions are generally undertaken post-intervention. These provide significant insights into understanding the lived experiences of each woman and the impact of the intervention as a whole, compared to the narrower lens of pre-selected quantitative outcome measurements. Mannell and Davis [23] argue that qualitative methods have been underutilised in health research. Specifically, researchers are reluctant to utilise qualitative methods due to the perception of diminished robustness and associated publishing bias, which has resulted in these methods being omitted following pre-trial phases.

As articulated by Deaton and Cartwright [24] the RCT design does not guarantee an accurate assessment of average treatment effects nor diminish the need to consider the influence of covariates. Further, these authors highlight the importance of combining methods to build scientific knowledge. There are several plausible solutions to rectify this problem via a change to the methodological design. When appropriately implemented, a mixed method research design combining quantitative (e.g. RCT, quasi-experimental) and qualitative methods (e.g. individual or focus group interviews) can significantly enhance the depth of study findings [25]. Furthermore, implementing suggested changeCs (identified during qualitative analyses) can overcome the limitations of a RCT by assessing the individual and contextual factors which determine the potential transferability of an intervention into clinical practice [25]. We believe that actioning consumer input is the key to implementing appropriate study methodologies to assess behavioural interventions.

In recent years, the importance of consumers in research has become apparent, with many funding bodies, such as Australia's National Health and Medical Research Council (NHMRC), now requiring consumer input into study design, governance and outcome interpretation [26]. The WWACP has worked with consumers extensively over the last decade to refine both the WWACP intervention as well as the study methodologies used to assess the intervention. For example, previous participants are quoted as saying *“[I] Was in control [group] so no benefit this far …*” and *“Being a participant in the control group was difficult and I had to withdraw. There needs to be better support after active treatment has finished.”* As demonstrated by these quotes, controlling who does and does not receive the intervention has a negative impact on participants (and on study retention). As clinical researchers working in clinical spaces with women with complex needs, it is our duty to provide them with the best possible, scientifically credible interventions [27]. From our previous study iterations, we know the WWACP intervention improves QoL for women after breast and gynaecological cancer treatment. When participants are randomly allocated to the control group, they receive what is considered ‘standard care’ (i.e. expected routine follow up from their treating healthcare team). Are we then depriving them of best possible outcomes [28]? To address our duty of care towards our control participants, we have always offered access to the intervention resource materials at study completion. Yet there is a methodology-robust alternative.

Although individuals are made aware of the chance of allocation to a control group with no intervention (in the case of our studies a 50 % chance) this threatens the integrity of the research process – once women realise they do not receive the intervention, they often withdraw from the study altogether before longitudinal assessments can be completed. In this scenario other study designs, such as non-randomised pre-post or waitlist controlled designs could be more ethical [28]. We also have advocated (and received competitive funding for) several single arm studies of the intervention, using more contemporary methods such as interrupted time series, which ensures all participants receive the intervention or act as their own controls. These non-randomised studies [29] are perceived to be more ethical as each participant receives the individualised intervention. Regarding the WWACP, this is especially relevant as management of lifestyle factors is recommended for all individuals who have finished treatment for cancer [30]. Alternatively, if randomization is still desired by the research team for cogent methodological reasons, waitlist controlling is implemented. In the future, and where the research question warrants it, A/B testing methodology will also be employed. A/B testing involves the random allocation of participants to separate groups, with the participants in each group receiving a different version of the intervention [31]. In the case of the WWACP and subsequent iterations, one group could receive the lifestyle intervention materials and concurrent nurse-led virtual consultations, whilst the other group only receives the lifestyle intervention materials (similar to, however more comprehensive than, standard care). This could ensure each participant is offered at least part of the intervention whilst enabling comparisons in intervention efficacy. However, alternative designs, such as those incorporating waitlist controls, could introduce new challenges related to data and its interpretation that would need to be addressed (e.g. unbalanced stages, potential biasing of the true effect of different active treatments [32,33]). While these designs might appear to offer a solution, they are not a guaranteed fix for obtaining a robust assessment of the effectiveness of the behavioural intervention.

In conclusion, the limitations of randomized-controlled trials (RCTs), particularly their misalignment with real-world outcomes, highlight the need for more person- and condition-relevant evaluation methods. The authors found that RCTs often failed to capture the complexity of individual experiences, potentially leading to interventions that might not translate effectively outside controlled settings. In our opinion, while RCTs provide valuable data, they often overlook the nuances of consumer needs and preferences, which are essential for ensuring practical relevance. By incorporating consumer input, trial designs could be refined to align interventions with real-world contexts, fostering more holistic and effective solutions. For multi-faceted behavioural interventions, the authors have observed a mixed method approach offers a more comprehensive evaluation of both efficacy and implementation potential. A non-randomized pre-post study design might be more ethical, ensuring maximal intervention delivery and participant benefit. If randomization is required, A/B or waitlist control designs can help address ethical concerns. Together with consumer feedback, these methods provide a more balanced framework for assessing interventions.

## CRediT authorship contribution statement

**Sarah M. Balaam:** Writing – review & editing, Writing – original draft, Data curation, Conceptualization. **Alexandra L. McCarthy:** Writing – review & editing, Conceptualization. **Natalie K. Vear:** Writing – review & editing, Writing – original draft, Conceptualization. **Mackenzie J. Petie:** Writing – review & editing, Conceptualization. **Debra J. Anderson:** Writing – review & editing, Conceptualization. **Janine P. Porter-Steele:** Writing – review & editing, Writing – original draft, Data curation, Conceptualization.

## Consent to participate

Informed consent was obtained from all individual consumer participants included in the study from which quotes were sourced.

## Ethics approval

Participant quotes used for this paper were sourced from a study which was performed in line with the principles of the Declaration of Helsinki. Approval was granted by the Uniting*Care* Health Human Research Ethics Committee (Date February 05, 2021/No 202103).

## Funding sources

This work was supported by the Wesley Research Institute grant (Grant number ID2020CR02). The funding body was not involved in the study design; in the collection, analysis, and interpretation of data; in the writing of the report; or in the decision to submit this article for publication.

## Declaration of competing interest

The authors declare the following financial interests/personal relationships which may be considered as potential competing interests: Janine P. Porter-Steele reports financial support was provided by Wesley Research Institute. If there are other authors, they declare that they have no known competing financial interests or personal relationships that could have appeared to influence the work reported in this paper.
